# An Edible Antibacterial Coating Integrating Lytic Bacteriophage Particles for the Potential Biocontrol of *Salmonella enterica* in Ripened Cheese

**DOI:** 10.3390/polym16050680

**Published:** 2024-03-02

**Authors:** Marta M. D. C. Vila, Edjane C. Cinto, Arthur O. Pereira, Denicezar Â. Baldo, José M. Oliveira Jr., Victor M. Balcão

**Affiliations:** 1 VBlab—Laboratory of Bacterial Viruses, University of Sorocaba, Sorocaba 18023-000, SP, Brazil; ediconsorte@gmail.com (E.C.C.); 00092394@aluno.uniso.br (A.O.P.); victor.balcao@prof.uniso.br (V.M.B.); 2 LaFiNAU—Laboratory of Applied Nuclear Physics, University of Sorocaba, Sorocaba 18023-000, SP, Brazil; denicezar.baldo@prof.uniso.br (D.Â.B.); jose.oliveira@prof.uniso.br (J.M.O.J.); 3 Department of Biology and CESAM, University of Aveiro, Campus Universitário de Santiago, P-3810-193 Aveiro, Portugal

**Keywords:** lytic bacteriophage particles, *Salmonella enterica*, antibacterial edible biopolymeric coating (EdiPhage), bacteriophage structural and functional stabilization

## Abstract

The goal of this research was to create an antibacterial biopolymeric coating integrating lytic bacteriophages against *Salmonella enterica* for use in ripened cheese. *Salmonella enterica* is the main pathogen that contaminates food products and the food industry. The food sector still uses costly and non-selective decontamination and disease control methods. Therefore, it is necessary to look for novel pathogen biocontrol technologies. Bacteriophage-based biocontrol seems like a viable option in this situation. The results obtained show promise for food applications since the edible packaging developed (EdiPhage) was successful in maintaining lytic phage viability while preventing the contamination of foodstuff with the aforementioned bacterial pathogen.

## 1. Introduction

Foodborne illnesses, or foodborne diseases, are a major cause of morbidity and mortality and a major public health problem worldwide. According to estimates from the World Health Organization (WHO), eating food tainted with dangerous microorganisms results in the deaths of 1.9 million children annually [1,2]. The three primary bacteria that cause foodborne infections are *Salmonella enterica*, *Escherichia coli*, and *Staphylococcus aureus* [3,4].

Over 50% of the recognized serotypes of Salmonella are caused by the *Salmonella enterica* species, which is responsible for most human infections with the bacteria [5,6]. Daniel Salmon identified and described Salmonella, a bacillus Gram-negative member of the Enterobacteriaceae family, in 1885 [5]. Salmonella is typically split into two species: *Salmonella enterica* and Salmonella bongori. There are over 2500 recognized serotypes of Salmonella. The contamination of food by Salmonella occurs through various factors, such as exposure time to the environment during the manufacturing process, preparation, and/or storage [7]. Salmonella is the most prevalent foodborne pathogen causing more than 93 million cases of salmonellosis and 150,000 fatalities every year [5]. These bacillus outbreaks in recent years have been linked to a variety of items, including raw tuna, cabbage, chicken, eggs, pistachios, cucumbers, and pre-cut melons [4]. Many proposals have been evaluated to control the main pathogens causing food poisoning. The developing technologies are anticipated to be sustainable and to have the least negative effects on nutrients and food quality, all the while taking into account the difficulties of effectively inactivating pathogenic microorganisms in various food matrices [8].

Contamination by bacteria can occur during the slaughter, milking, fermentation, processing, storage, or filling among other processes [7]. The increasing demand for high-quality, shelf life-extended, ready-to-eat food products has led to the development of new processing technologies that ensure that the product’s natural attributes and appearance are not significantly compromised [9].

Among the various strategies used to minimize the microbial load of some foods, the use of antibiotics has been explored [6]. However, antibiotic substances present restricted use due to both the negative impact on human antimicrobial therapies as well as the selection of more resistant microorganisms [10]. The use of physical methods, such as superheated steam, dry heat, and UV light, can lead to product acceptability problems and the deterioration of the organoleptic properties of foods [11,12]. In addition, some approaches often used in processed foods to reduce contamination by foodborne pathogens cannot be directly applied to fresh fruits, vegetables, and ready-to-eat products [13]. New processing technologies such as gamma-ray irradiation, plasma processing, high-pressure processing, pulsed electric field, and ultrasound, can be efficient, but have high costs [14]. Therefore, the development of new processing strategies to reduce bacterial pathogens in food while still meeting consumer demands for minimally processed foods, with low concentrations of chemical preservatives, has been more and more urgent [1].

In this context, bacteriophages (or phages) have emerged as a bacterial biocontrol tool with enormous potential in the fight to reduce the burden of infectious diseases [2,14]. Bacteriophages were discovered in the mid-1910s by British scientist Frederick Twort [15]. During his research with virus cultivation, Twort realized that plates contaminated with some bacteria showed zones of lysis, and therefore, he assumed that there was possibly some microorganism capable of lysing bacterial cultures. The official discovery was made in 1917 by the French-Canadian microbiologist Felix D’ Herelle, who used lytic viruses from *Shiguella dysenteriae* to treat his dysentery [15]. D’ Herelle named this virus a bacteriophage (or phage) and was congratulated as the “father” of modern virology [10]. Bacteriophages are viruses that solely and exclusively infect susceptible bacterial cells and have no metabolic machinery of their own, hence being intracellular parasites requiring a bacterial host cell to replicate [16,17,18]. Many applications of bacteriophages in the control of foodborne pathogens have been proposed over the years, with relative success [14]. Bacterial biocontrol using bacteriophages has the unique advantage that phage particles are natural antibacterial agents, self-multiplying and highly specific [16]. Another interesting property is the remarkable stability of bacteriophages in foodstuff [11]. Furthermore, according to Sillankorva et al. [19] and Sahu et al. [16], bacteriophages have several other advantages as biocontrol agents in foodstuff, including (but not limited to): (i) specificity to reaching their bacterial host cells while keeping the local microbiota unaffected; (ii) self-replication and self-limitation, such as in multiplying while the target host cells are still present and viable; (iii) adaptation to the defense mechanisms of the bacterial cells; (iv) a very low inherent toxicity, since they are formed basically of nucleic acids and proteins; (v) a very low cost of isolation and simplicity of handling; and (vi) tolerance to various food conditions. In this way, researchers have therefore attempted to employ these bacterial viruses to combat a variety of bacterial illnesses in humans and animals [5,16]. Phage particles can be used to battle foodborne pathogens at every stage of manufacturing across the food chain. Bacteriophages are appropriate in (i) stopping or lessening illness and colonization in livestock; (ii) cleaning up carcasses and other unprocessed goods like fresh produce, eggs, and fruits; (iii) the decontamination of surfaces and equipment; and (iv) increasing the shelf life of perishable industrialized foods [7,20].

Keeping in mind everything described above, the main objectives of the research presented were to isolate lytic bacteriophages for *Salmonella enterica* from ambient sources and to characterize them from both physicochemical and biological points of view, aiming at producing an edible biopolymeric film integrating a phage cocktail containing the isolated phages with the potential for controlling *Salmonella enterica* in foodstuff, using matured cheese as a food matrix model.

## 2. Materials and Methods

### 2.1. Materials

#### 2.1.1. Preparation of Culture Media, Solutions, and Laboratory Materials

All culture media and solutions intended for microbiological procedures, together with materials such as microtubes, test tubes, Falcon tubes, tweezers, micropipette tips, among others, were sterilized in a Prismatec^®^ CS-A line vertical autoclave (Prismatec Indústria e Comércio, Itu, SP, Brazil) for 30 min at 121 °C. Tap water was purified in a Master System All (model MS2000, Gehaka, São Paulo, SP, Brazil) to a final resistivity of ca. 18.18 MΩ·cm and conductivity of 0.05 µS·cm^−1^, and was used in the preparation of all media and solutions. Media and reagents were weighed on an analytical scale from Marte Científica^®^ (model OHAUS AS200S, Santa Rita do Sapucai, MG, Brazil). A biological safety cabinet (model Filterflux^®^ Class II B2, SPLab, Piracicaba, SP, Brazil) was used for manipulations with bacteria, bacteriophages, culture media, and microbiological activity measurements.

#### 2.1.2. Biological Materials

Cefar Diagnóstica (São Paulo, SP, Brazil) provided the *Salmonella enterica* CCCD-S004 strain collection bacterium used as a bacterial host.

Phage SentS01L was isolated from lake water, whereas phage SentS01T was isolated from a soil sample, both collected in the surroundings of the Veterinary Hospital of UNISO, Sorocaba, SP, Brazil (geographic coordinates: 23°29′58.7″ S; 131 47°23′45.2″ W).

#### 2.1.3. Chemicals

The reagents were purchased from (i) Dinâmica Química Contemporânea Ltd. (Diadema, SP, Brazil) (anhydrous dibasic sodium phosphate, monobasic sodium phosphate, calcium chloride, and sodium chloride); (ii) Sigma-Aldrich (St. Louis, MO, USA) (culture media Tryptic Soy Agar (TSA) and Tryptic Soy Broth (TSB), polyethylene glycol (PEG) 8000, d-gluconolactone (GDL), magnesium sulfate, Trizma hydrochloride (Tris-HCl), sodium alginate, and uranyl acetate); (iii) Gibco Diagnostics (Madison, WI, USA) (microbiological solid agar); (iv) Merck-Millipore (Darmstadt, Germany) (sterilizing filtration systems/Stericup™-GP); (v) Labsynth (Diadema, SP, Brazil) (magnesium sulfate); (vi) Anidrol (Diadema, SP, Brazil) (calcium carbonate (CaCO_3_)); and (vii) BioRad (Santo Amaro, SP, Brazil) (disruption buffer, molecular weight markers, and Coomassie Brilliant Blue G-250).

### 2.2. Experimental Procedures

#### 2.2.1. Preparation of a Suitable Bacterial Suspension of *Salmonella enterica* CCCD-S004

The host bacteria were incubated at 37 °C for 12 h in solid TSA and were hydrated after in TSB liquid medium.

#### 2.2.2. Bacterial Lawns (*Salmonella enterica* CCCD-S004) Using the Pour Plate Technique

For the preparation of host bacterial lawns, 100 µL of bacterial suspension were added in a tube with 5 mL of molten top agar-TSB (MTA-TSB). After homogenization, these samples were poured into plates with solid TSA, allowed to dry out, and then incubated at 37 °C during 12 h.

#### 2.2.3. Spot Test Verification of Lytic Activity in the Two Phage Suspensions

For the verification of the lytic activity of the isolated phages, 10 µL of each phage suspension were poured onto host bacterial lawns and incubated overnight at 37 °C. After this time, clear lysis zones, which are a sign of the existence of lytic bacteriophages, could be seen.

#### 2.2.4. Phage Virion PEG-Precipitation

In a sterile mixture of polyethylene glycol (PEG) 8000 (10%, *w*/*w*) and NaCl (1 mol/L) (2:1), phage suspension samples were added. These suspensions were incubated at 4 °C during 12 h and, after this time, the samples were centrifuged (11,000 rpm, 4 °C, 45 min). The pellet was resuspended in 5 mM MgSO_4_, and the supernatant discarded.

#### 2.2.5. Bacteriophage Enumeration

In accordance with Adams [18], the bacteriophage titer was determined. Serial dilutions were prepared employing stock bacteriophage suspension (50 µL) and SM phage buffer (450 µL) (200 mM NaCl; 10 mM MgSO_4_; and 50 mM Tris-HCl in pH 7.5). In sequence, 50 µL of each dilution was added to 100 µL of bacterial culture grown and 4 mL of MTA-TSB. The suspension was added to a TSA Petri plate and incubated at 37 °C for 12 h. Following this time frame, lytic plaques were seen in each dilution and counted taking into account dilutions containing 20–200 bacteriophage plaques. Next, the bacteriophage titer (PFU/mL) was computed as number of phage plaques formed×1dilution×1Vbacteriophage inoculum(mL). The resulting phage titers were 1.40 × 10^12^ PFU/mL (phage SentS01L) and 1.72 × 10^12^ PFU/mL (phage SentS01T).

#### 2.2.6. Transmission Electron Microscopy (TEM) Analyses

The phage particles were centrifuged in a micro-ultracentrifuge from Beckman-Coulter (model Optima TLX, Indianapolis, IN, USA) for 150 min at 45,000 rpm and 4 °C. The samples were negatively stained employing uranyl acetate at 2% (*w*/*v*) and pH 7.0, [21,22] and photomicrographed in a Transmission Electron Microscope from JEOL (model JEM 2100, Tokyo, Japan) using a high-resolution CCD camera from GATAN Inc. (model ORIUS™ 832.J4850 SC1000B, Pleasanton, CA, USA). The software Gatan Microscopy Suite (DigitalMicrograph from GATAN Inc., version 2.11.1404.0) was used to obtain digital photos of the phage virions.

#### 2.2.7. Sodium DodecylSulphate PolyAcrylamide Gel Electrophoresis (SDS-PAGE) Analysis of Phage Virion Structural Proteins

SDS-PAGE was used for determining the molecular weights of the bacteriophage SentS01L and SentS01T structural proteins, employing a Mini-PROTEAN^®^ Tetra Cell from Bio-RAD (Hercules, CA, USA). In an Eppendorf, 500 µL of disruption buffer was added to each sample of bacteriophage suspension, and the mixture was boiled for ten minutes. After, 20 µL of sample supernatant and 5 µL of molecular weight markers were applied to the 5% acrylamide–bisacrylamide concentration gel/12% acrylamide–bisacrylamide separation gel, and electrophoresis was run for 60 min. Coomassie Brilliant Blue G-250 was used to dye the gel, after which it was photographed in high resolution for further analysis.

#### 2.2.8. Formulation and Characterization of the Edible Antibacterial Coating (Ediphage) Integrating the Lytic Phage Cocktail

*Formulation of the EBP Film*. The EdiPhage formulation (Table 1) was prepared via internal gelification as described by Balcão et al. [23,24] and Łętocha et al. [25]. The internal gelification method was used to polymerize alginate at room temperature for 72 h. To initiate alginate polymerization, a fresh aqueous δ-gluconolactone (GDL) solution was used to release of the calcium ions dispersed in the formulation. Calcium carbonate (CaCO_3_) was employed as a source of calcium ions. The previously made polymeric dispersion was homogenized with this hydrolysis solution, and the cheese samples were dipped in it. Following this period, the cheese matrices were kept at 4 °C until further analyses were in order.

The appropriate phage virion/bacterial cell ratio (i.e., Multiplicity Of Infection—MOI), aiming at the inactivation of host bacteria (viz. MOI 100 and MOI 1000), was used based on a previous work by Pereira et al. [21].

*EdiPhage Thickness*. The average thickness of the polymerized coatings was determined using a caliper with a resolution of 0.001 mm, through five random measurements on the area of each EdiPhage.

Evaluation of the Maintenance of the Lytic Viability of the Bacteriophage Particles Integrated in the Edible Antibacterial Coating. A sample of EdiPhage was removed from a cheese matrix sample and positioned in the center of a bacterial lawn of *Salmonella enterica* CCCD-S004. After incubation for 24 h at 37 °C, a macroscopical analysis was carried out to observe the presence of clear zones of lysis in the bacterial lawn surrounding the EdiPhage samples.

*Evaluation of the Potential for Cytotoxicity of the Ediphage*, *Via Disc Diffusion Assay*. The disc diffusion assay employing cell lines of immortalized human keratinocytes (HaCaT) and mouse fibroblasts (3T3) [26,27,28] was used for the evaluation of the cytotoxicity potential of the EdiPhage integrating the cocktail of lytic phages. A Petri plate containing the cells was filled with a small disc of sample (EdiPhage) and was incubated for 24 h at 37 °C in a 5% CO_2_ atmosphere. The same protocol was used for a negative control (a disc of innocuous paper) and positive control (latex). The presence of cytotoxicity was detected by the formation of a transparent halo due to cell lysis surrounding the sample tested [29].

*Determination of the Ediphage Elemental Composition* Via *Energy Dispersive X-ray Fluorescence (EDXRF) Analyses*. An X-ray fluorescence spectrometer with energy dispersion (EDXRF) from Amptek (Bedford, MA, USA) was used for the determination of the elemental composition of the EdiPhage formulations. Every measurement was performed with ambient air, and each sample’s measuring time was fixed at 300 s (live time).

*Fourier Transform Infrared Spectrophotometry (FTIR) Analyses*. A Fourier Transform Infrared Spectrophotometer from Agilent (model Cary 630, Santa Clara, CA, USA) was used for the FTIR spectra of EdiPhage samples. The measurements were obtained in the range from 4000 cm^−1^ to 400 cm^−1^ and a resolution of 2 cm^−1^.

*Thermal Analyses Via Differential Scanning Calorimetry (DSC)*. Shimadzu’s DSC-60 microcalorimeter (model DSC-60, Kyoto, Japan) was used to conduct DSC analyses combined with a Thermal Analyzer TA 60W (Shimadzu, Kyoto, Japan). The parameters used were a temperature increase from ca. 25 °C up to 300 °C, at a heating rate of 10 °C min^−1^, under an inert atmosphere (argon of 50 mL min^−1^ [26]. The samples weighed 1.080 mg (plain EdiPhage) and 1.820 mg (bioactive EdiPhage).

*Tomographic Analyses Via X-ray Transmission (XRT)*. An X-ray transmission tomograph from Bruker microCT (model SkyScan 1174, Kontich, Belgium) was employed for tomographic images. The software NRecon™ from Bruker (version 1.6.9.4, Kontich, Belgium) used the algorithm of Feldkamp et al. [30] in the process of reconstructing the tomographic images. The software CTVox™ (version 2.6.0 r908-64 bit, from Bruker microCT), CTan™ (version 1.13.5.1-64 bit, from Bruker microCT) and CTvol (version 2.2.3.0-64 bit, from Bruker microCT) were used for the processing of the tomographic images.

*Scanning Electron Microscopy Analyses (SEM)*. The images of the EdiPhage sample were obtained using a scanning electron microscope (JEOL, model JSM-IT200, Tokyo, Japan), with an Energy Dispersive X-ray Spectrometer (EDS) detector (JEOL, model DRY SD™25 Detector Unit, Tokyo, Japan). The sample coatings were prepared through the cathodic pulverization of Au (92 Å thickness) in a metalizing device.

*Mechanical Resistance Properties*. A texturometer from Stabile Micro Systems (model TA-TX Plus, Godalming, UK) was used to assess the mechanical qualities of the EdiPhage, employing a maximum force of 5 kg and a distance of 5 mm. All experiments were performed in triplicate, using sample dimensions of 3 cm × 2 cm.

#### 2.2.9. Statistical Analyses

The statistical analysis of the data was carried out utilizing GraphPad Prism 7.04 software (GraphPad Software, San Diego, CA, USA). The data’s normal distribution was examined using a Kolmogorov–Smirnov test. The homogeneity of variance was assessed using Levene’s test. The significance of bacterial concentrations was tested using two-way ANOVA and the Bonferroni post-hoc test. A value of *p* < 0.05 was considered to be statistically significant.

## 3. Results and Discussion

### 3.1. Morphological Characteristics of the Bacteriophage Virions Via TEM Analyses

**Figure 1:**
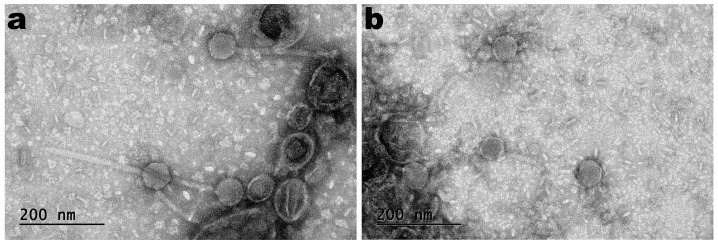
Negative staining TEM photomicrographs of phages SentS01L (**a**) and SentS01T (**b**).

TEM photomicrographs of phages SentS01L and SentS01T are displayed in Figure 1.

The phages’ morphology (Figure 1) indicates that both phages exhibited siphovirus morphotypes and were classified under the Caudoviricetes class. Phages SentS01L and SentS01T displayed flawless icosahedral heads and lengthy, flexible tails that were not contractile.

### 3.2. Structural Protein Profile of Phage Sents01l and Sents01t Virions Obtained through SDS-PAGE Analysis

**Figure 2:**
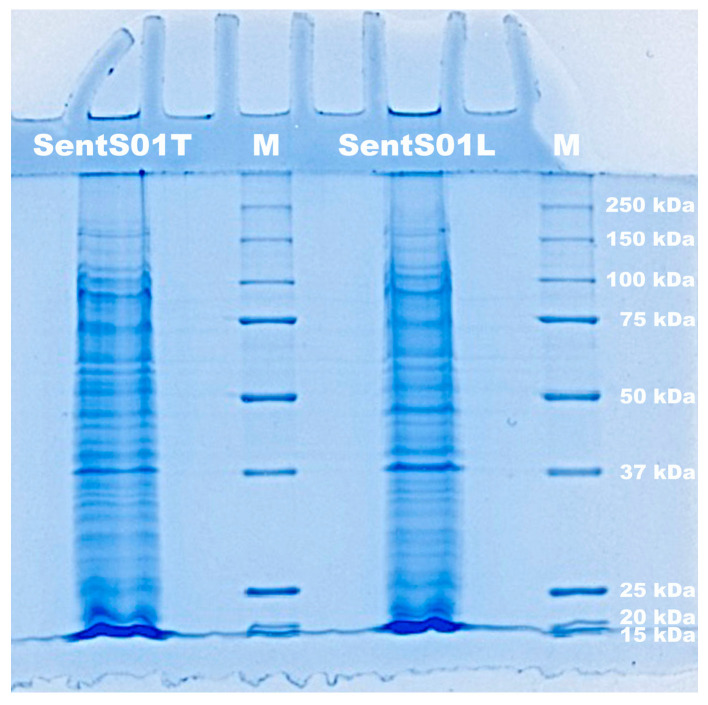
Electrophoretogram of the proteins of phages SentS01L and SentS01T and of molecular weight markers (lane M).

The results of SDS-PAGE electrophoresis (Figure 2) show that both phages had a structural protein profile across a broad range of molecular weights.

### 3.3. Preparation and Characterization of the Edible Antibacterial Coating (Ediphage) Integrating the Lytic Bacteriophage Cocktail

The edible antibacterial coating was prepared with sodium alginate via ionotropic gelling. Alginate is a polysaccharide of natural origin, which shows interesting characteristics, including its non-toxicity, biodegradability, and gelling ability [31]. Hence, it was chosen for the formulation of the edible antibacterial coating. Sodium alginate has been effectively used in edible coatings and films to protect food as well as to serve as a carrier for certain food preservation agents (antioxidants and antimicrobials) [32]. Moreover, additional research has suggested that matrices based on alginate are appropriate for phage inclusion [33]. Using CaCO_3_ in the alginate solution, as it is poorly soluble, allowed a controlled and uniform gel formation.

**Figure 3:**
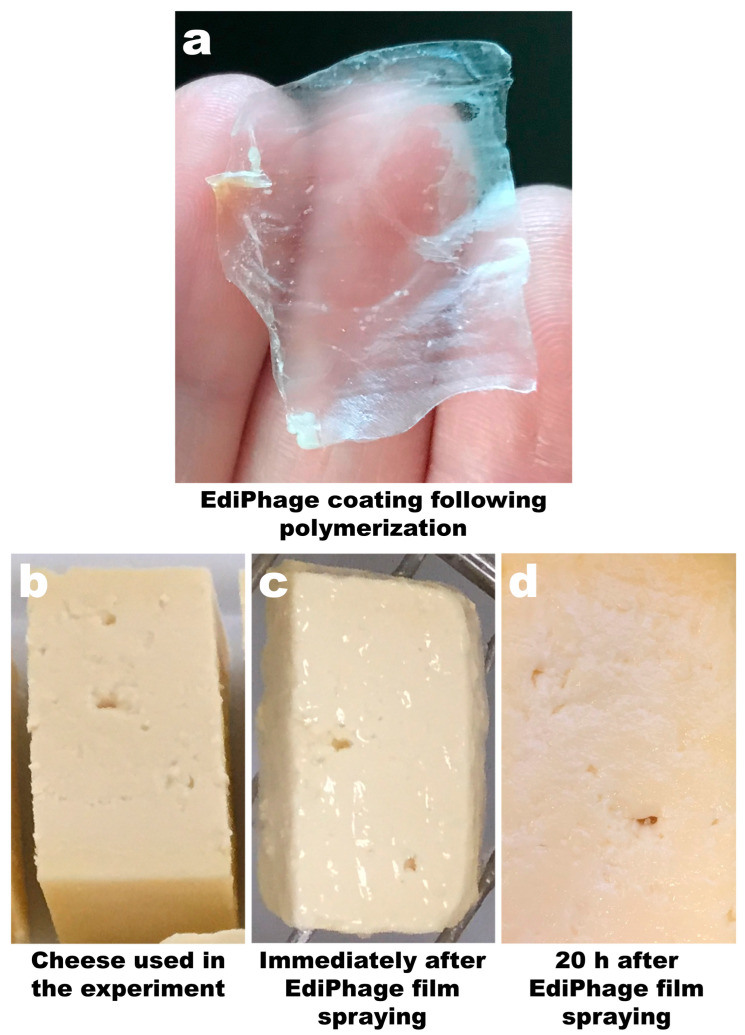
Image of the calcium alginate EdiPhage obtained via ionotropic gelling (**a**) and images of ripened cheese samples before spraying with the EdiPhage formulation (**b**), immediately after spraying with the EdiPhage formulation (**c**), and 20 h after spraying with the EdiPhage formulation (**d**).

The technique allowed for the preparation of a coating with a film-like appearance, thickness, and flexibility suitable for use as an antibacterial coating for food applications. The coating was uniformly translucent and allowed the formation of a delicate film on the cheese samples (Figure 3a,c) with a thickness of 0.03 mm ± 0.009 (EdiPhage coating) and 0.03 mm ± 0.005 (phage-free coating). After a period of 20 h, the film remained unchanged, indicating that the addition of the phage cocktail did not alter the three-dimensional structure of the gel (Figure 3d).

Since phage particles’ availability and/or vitality might be impacted by their immobilization on various matrices, the activity of the phage virions immobilized in the EdiPhage coating was assessed. The process of integrating them into films, coatings, and hydrogels exposes them to stress conditions including stirring, drying, and mixing [34].

**Figure 4:**
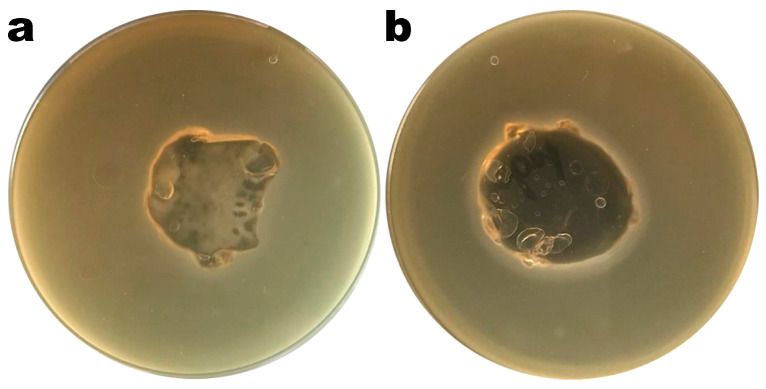
Results from assessment of the lytic viability of entrapped bacteriophage particles within the polymerized EdiPhage coating. (**a**) EdiPhage matrix devoid of phage particles and (**b**) bioactive EdiPhage coating integrating the phage cocktail at MOI 1000.

Images of Petri plates with *S. enterica* CCCD-S004 and samples of the Ediphage coating with and without the cocktail of lytic phages are shown in Figure 4a,b. Lysis zones are visible in Figure 4b, suggesting that the phage cocktail’s lytic activity is maintained after the EdiPhage formulation polymerizes. The phage virion particles’ entrapment within the EdiPhage coating’s polymerized calcium alginate biopolymeric matrix made possible the virions’ structural and functional stability while preserving their lytic viability.

**Figure 5:**
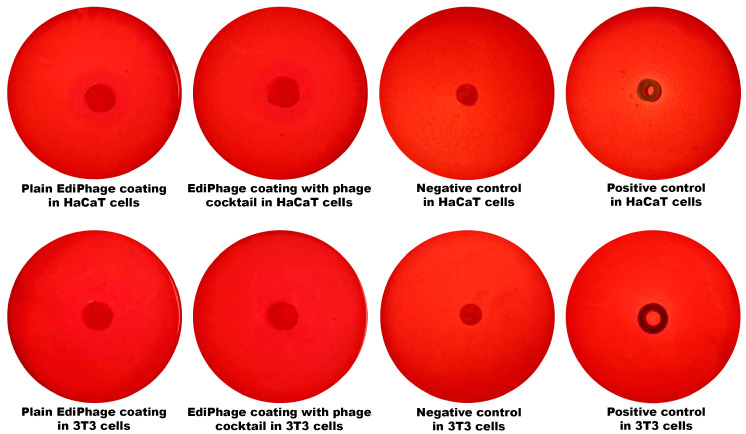
Analysis of the cytotoxicity with HaCaT and 3T3 cell lineages of the antibacterial EdiPhage coating integrating the cocktail of lytic phage particles.

Regarding the assessment of the cytotoxicity of the bioactive antibacterial EdiPhage coating, the results indicate that there was no cell death whatsoever caused by contact with the EdiPhage coating after 24 h in the assay performed with either HaCaT or 3T3 cell lines (Figure 5).

**Figure 6:**
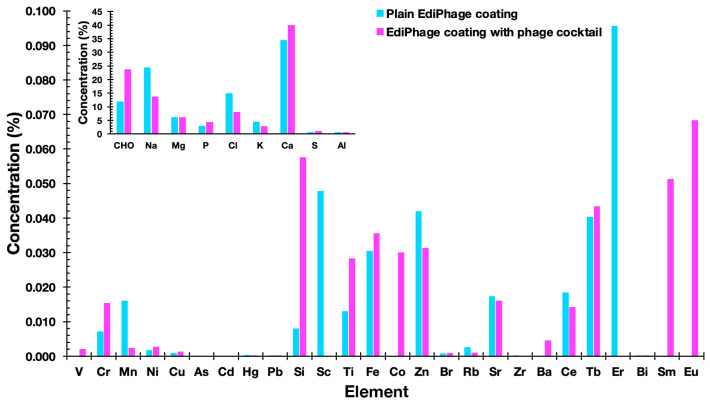
Elemental profiles of the plain EdiPhage coating and of the antibacterial bioactive EdiPhage coating integrating the phage cocktail, obtained via EDXRF.

The elemental compositions of the EdiPhage coatings devoid of bacteriophage cocktail and integrating the bacteriophage cocktail were determined using an X-ray fluorescence spectrometer. The most common substances were cellulose [(C_12_H_20_O_10_)_n_], sodium (Na), chlorine (Cl), and calcium (Ca) (Figure 6). Sodium alginate, the basis of the edible coating, is extracted from seaweed, and, in its extraction and purification process, formaldehyde, 0.2% HCl solution and 2% sodium carbonate solution are normally added [35]. Therefore, cellulose, sodium, and chlorine—in larger amounts—are derived, most likely from sodium alginate. Because calcium (Ca) was added during the ionotropic gelation process, it was also discovered in larger concentrations. Phosphorus (P), potassium (K), and magnesium (Mg) were additional notable elements, most likely originating from the phage suspensions. But these substances are not thought to be harmful. The other elements present, but in lower concentrations, probably originated from the raw materials used to obtain the EdiPhage coating. The elements may be present naturally, purposefully added during the synthesis of the product, or accidentally introduced as a result of interactions with processing machinery throughout the manufacturing process [36].

**Figure 7:**
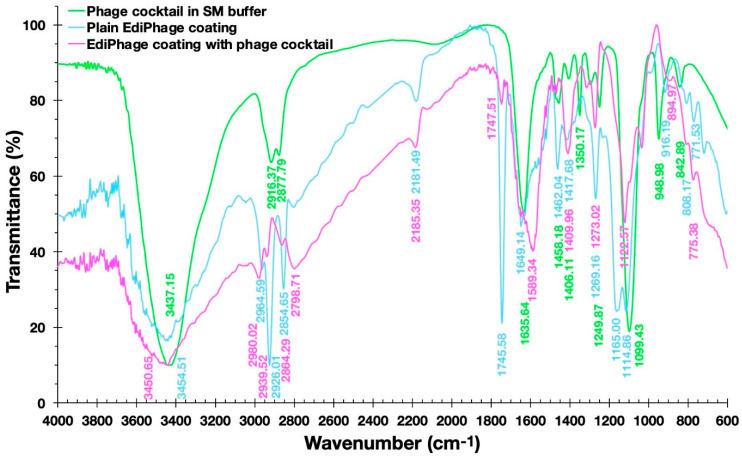
Fourier-transform infrared (FTIR) spectra of the plain EdiPhage coating (blue line), of the antibacterial bioactive EdiPhage coating integrating the phage cocktail (magenta line), and of the phage cocktail in SM buffer (green line).

Figure 7 shows the FTIR spectra of the plain EdiPhage coating, the antibacterial bioactive EdiPhage coating integrating the phage cocktail, and the phage cocktail in SM buffer.

Fernandes et al. [37], analyzing a sodium alginate-based polymer, observed a broad band between 3200 and 3600 cm^−1^ corresponding to the elongation of the OH groups present in the alginate polymer chain. The same was observed by Daemi and Barikan [38] between the bands between 3000 and 3600 cm^−1^ (Figure 7). In the spectra obtained, wide bands around 3500 cm^−1^ can also be observed. Fernandes et al. [37] also observed bands at 1414 cm^−1^ and 1621 cm^−1^, which were correlated, respectively, with the asymmetric and symmetric axial deformations of the -COO- groups, indicating the presence of a carboxylic acid group in the alginate. In the studied spectra, more intense peaks were observed in the plain EdiPhage coating (without phage particles) in the approximate range between 1700 and 1100 cm^−1^, particularly in the peaks at 1745.58 and 1165.00 cm^−1^ (Figure 7). Perhaps there could be some kind of interaction between the compounds arising from the SM buffer and the -COO- groups. Helmiyati and Aprilliza [39] observed a peak around 2928 cm^−1^ in an alginate sample and indicated that it was due to the stretching of the CH_2_ group. A close peak (2926.01 cm^−1^) was observed in the plain EdiPhage coating (without phage particles) (Figure 7). The absence of this peak was found in the antibacterial bioactive EdiPhage coating integrating the phage cocktail (Figure 7), which might indicate some kind of interaction between the compounds arising from the SM buffer and the alginate, such as CH-O bonding. Because of its very poor electronegativity when compared to oxygen and nitrogen, carbon is not considered a conventional traditional hydrogen bond donor. Nevertheless, several investigations have demonstrated that even aliphatic carbon atoms are capable of forming weak hydrogen bonds, which are designated as CH-O hydrogen bonds [40].

The bands occurred at 2964.59 and 2798.71 cm^−1^ (bioactive EdiPhage coating integrating the phage cocktail) and 2964.59; those at 2926.01 and 2854.65 cm^−1^ (plain EdiPhage coating (without phage particles)) can be attributed to aliphatic C-H vibrations [38]. These bands in the antibacterial bioactive EdiPhage coating integrating the phage cocktail were less evident, perhaps also due to CH-O bond formation. The presence of phage cocktail was irrelevant, probably related to the low percentage used to produce the EdiPhage coating.

**Figure 8:**
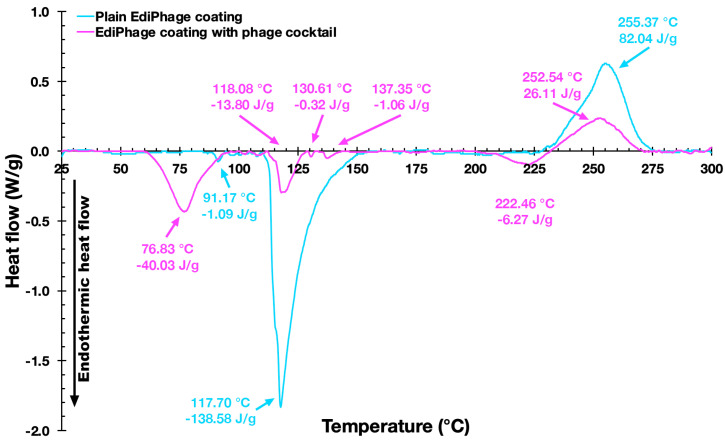
Differential scanning calorimetry thermograms of the EdiPhage coating devoid of phage particles (blue line) and of the EdiPhage coating integrating the cocktail of phage particles (magenta line).

In the characterizations of both the plain and bioactive EdiPhage coatings, thermal analyzes were also carried out. The DSC thermograms of the two EdiPhage polymeric coatings are displayed in Figure 8.

Figure 8 shows thermograms of the EdiPhage coating. The first endothermic events (sample and control) are probably related to coating dehydration [41]. The first endothermic events (76.67 °C/−35.79 J/g (sample) and 91.17 °C/−1.05 J/g control)) are probably related to the evaporation of hydration water molecules from the films [41]. The endothermic peak related to the dehydration process occurs at around 80 °C in sodium alginate [42]. The second endothermic events (118.08 °C/−8.41 J/g (sample) and 117.70 °C/−150.11 J/g (control)) may perhaps be due to a depolymerization process with a formation of carbonaceous residue [42,43]. The exothermic events (252.37 °C/49.43 J/g (sample) and 255.37 °C/80.91 J/g (control)) probably corresponded to cleavage enthalpies such as the breakage of bonds within the complex [44]. The sample also showed two small endothermic events, which might be due to the influence of components in the buffer solution (where the phages are diluted) that increase the conformational stability through the electrostatic interactions of the present components [45]. According to Helmiyati and Aprilliza [39], thermal analysis tests with alginates demonstrated that the decomposition temperature of pure sodium alginate was 251.12 °C, which is very close to the degradation temperature of the prepared coatings (sample and control), as can be seen in the exothermic events. From the results presented, it can be said that the coatings are stable for the intended application.

**Figure 9:**
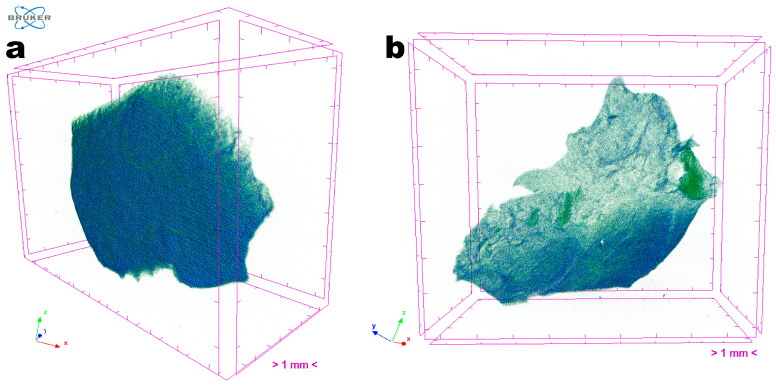
Images obtained through tomographic analyses via X-ray transmission of the EdiPhage coating loaded with phage particles: (**a**) front view of an EdiPhage sample and (**b**) slant view of the same EdiPhage sample. Three-dimensional image slices were gathered using an operating voltage set at 31 kV and electric current with 661 μA.

The three-dimensional images obtained via X-ray transmission tomography (XRT) (Figure 9) allow the uniformity and homogeneity of the EdiPhage coating to be observed. There is no zone of greater or lesser atomic density (Figure 9), in general, which is interesting for a food-grade coating.

**Figure 10:**
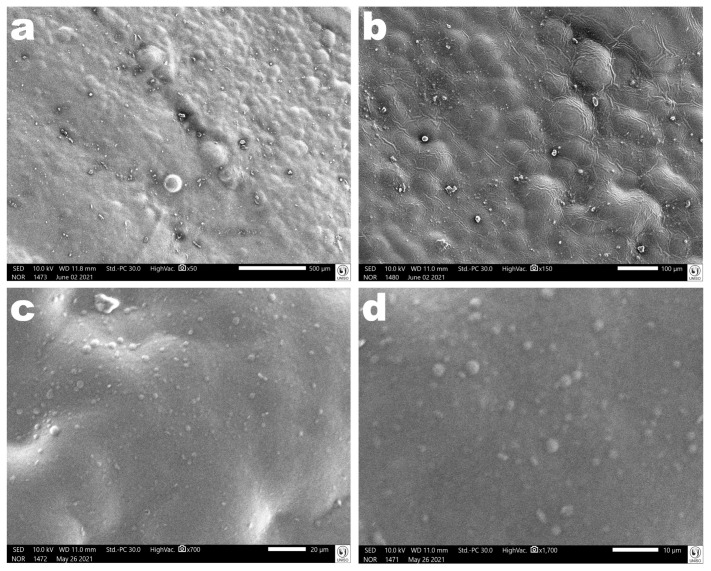
Photomicrographs of the polymerized antibacterial EdiPhage coating surface at several magnifications ((**a**): ×50, (**b**): ×150, (**c**): ×700, (**d**): ×1700). Images obtained via scanning electron microscopy (SEM) confirmed the formation of a coating with homogeneous characteristics.

Images obtained via scanning electron microscopy (SEM) of the surface of the polymerized antibacterial EdiPhage coating confirmed its homogeneous characteristics (Figure 10).

**Figure 11:**
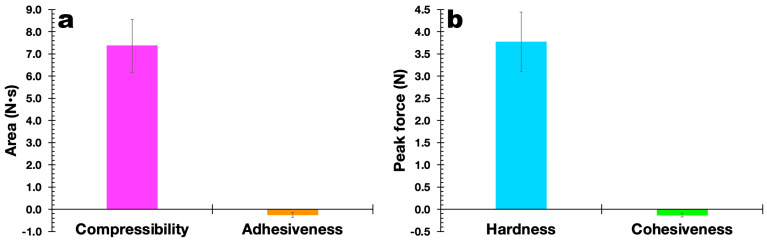
Results obtained for the compressibility and adhesiveness (**a**) and hardness and cohesiveness (**b**) of the antibacterial EdiPhage coating produced, integrating the cocktail of lytic bacteriophage particles.

The results obtained in the evaluation of the mechanical properties of the polymerized EdiPhage coating are displayed in Figure 11. In general, it can be said that the mechanical properties are directly linked to the degree of the crosslinking of the polymeric network, in addition to the characteristics of the alginate itself (molecular weight and M/G ratio (β-d-mannuronic acid (M blocks) and α-L-guluronic acid) (G blocks)) [46,47]. At the molecular level, the presence of Ca^2+^ ions is considered an obstacle to the rotation (movement) of alginate chains, decreasing their mobility and, consequently, the film’s ability to elongate [48]. Both the plain EdiPhage coating and the antibacterial EdiPhage coating integrating the cocktail of phage particles showed calcium contents close to each other. More elastic films have lower tensile strengths, with a consequent reduction in the maintenance of integrity [49]. Polysaccharide-based edible coatings and films are tough, highly soluble, colorless, and flexible because polysaccharides have linear structures. Edible coatings based on sodium alginate, normally, show good film-forming properties and are one of the most popular biodegradable polymers [50,51]. The mechanical properties are strongly influenced by the film formulation [52]. Accordingly, the range can be wide depending on the percentage of hydrocolloids added [53].

The results obtained allowed us to confirm the suitability of the formulation for the intended purpose. The hardness of the EdiPhage coating formulated was ca. 3.75 N, which is suitable for the intended use. A lower hardness makes the EdiPhage coating less brittle, and therefore, the chances of premature detachment from the surface of the product are low. Since the antibacterial EdiPhage coating is meant to package ripened cheese while allowing the release and mobility of the phage particles, the low value obtained for the adhesiveness of the coating endowed with antibacterial properties will exert a significant influence on the availability of the lytic phage particles in the intended application site. Given that the coating was created by polymerizing alginate polysaccharide, the greater value for the compressibility of the antibacterial EdiPhage coating was consistent with the formulation’s less-than-half-solid state. The release of phage particles at the product’s surface is crucial, and the poor cohesiveness generated (Figure 11) is consistent with the low adhesiveness value. Therefore, the outcomes in terms of (a not-so-high) hardness, higher compressibility, poor adhesiveness, and low cohesiveness are all consistent with the intended use of the antibacterial EdiPhage coating. Given that cohesiveness and adhesiveness are crucial qualities for food applications, the antibacterial EdiPhage coating’s capacity to adhere to the surface of the matured cheese, but not to a higher extent, is an extremely significant property. The capacity of the biopolysaccharide to establish bonds in polymer chains, which results in resistance to their separation when subjected to mechanical pressures, is primarily responsible for the mechanical qualities of the EdiPhage coating [54,55]. Therefore, the mechanical resistance criteria evaluated were compressibility, hardness, adhesiveness, and cohesiveness; resistance to traction, relaxation, and resilience properties were deemed irrelevant for the produced antibacterial EdiPhage coating. Hence, based on the data given here, the antibacterial EdiPhage coating can preserve its physical integrity.

## 4. Conclusions

Coatings/edible films obtained from vegetable polymers add important benefits to the product of its safety and quality. They can also be positively related to the growing trend of consumers searching for industrialized products that meet the “clean label” concept, that is, related to the use of organic ingredients, free of additives and artificial ingredients. Furthermore, the possibility of combining agents for biocontrol, such as phages, presents itself as a natural and green technology, effective for specifically targeting bacterial pathogens.

The antibacterial EdiPhage food coating showed adequate physicochemical characteristics, zero cytotoxicity, and maintenance of phage lytic activity against *Salmonella enterica*. In this sense, it can be stated that the antibacterial EdiPhage coating formulation has the potential for the biocontrol of *S. enterica* in ripened cheese.

However, it can be said that the development of edible films/coatings has occurred mainly at a laboratory scale, since there are still some limitations to be overcome, such as guaranteeing functional and organoleptic properties, attractive appearance, and compatible price, among other factors, and to guarantee the success of these materials on a commercial scale. The use of phages for biocontrol also requires further studies such as on evaluating the ability (or not) of phage penetration into food, the effect on phage survival in the face of abiotic factors, large volume production, etc.

## Figures and Tables

**Table 1:** Formulations of the edible antibacterial coating (EdiPhage) with (or without) phage particles.

Component		EdiPhage Film Formulation	
		Formulation 1 (Plain EdiPhage)	Formulation 2 (EdiPhage Integrating the Lytic Phage Cocktail)
Phage cocktail [% (*w*/*w*); m (mg)]	Phage SentS01L [% (*w*/*w*); m (mg)]	-	0.055; 55 µL
	Phage SentS01T [% (*w*/*w*); m (mg)]		0.055; 55 µL
Sodium alginate [% (*w*/*w*); m (mg)]		1.50; 1500	1.50; 1500
CaCO_3_ 22.5 mM [% (*w*/*w*); m (mg)]		0.1000; 100	0.1000; 100
δ-gluconolactone (GDL) 48 mM [% (*w*/*w*); m (mg)]		0.8600; 860	0.8600; 860
Ultrapure water [% (*w*/*w*); m (mg)]		97.54; 97,540	97.43; 97,430
TOTAL [% (*w*/*w*); m (mg)]		100; 100,000	100; 100,000

## Data Availability

Data are contained within the article.
